# Longitudinal construct validity of the minimum data set health status index

**DOI:** 10.1186/s12955-018-0932-9

**Published:** 2018-05-24

**Authors:** Aaron Jones, David Feeny, Andrew P. Costa

**Affiliations:** 1 Department of Health Research Methods, Evidence and Impact, McMaster University, 1280 Main St. W, Hamilton, ON L8S 4K1 Canada; 2 Department of Economics, McMaster University, 1280 Main St. W, Hamilton, ON L8S 4M4 Canada; 3 Department of Medicine, McMaster University, 1280 Main St. W, Hamilton, L8S 4K1 ON Canada

**Keywords:** Home care, Community care, Health-related quality of life, Longitudinal construct validity

## Abstract

**Background:**

The Minimum Data Set Health Status Index (MDS-HSI) is a generic, preference-based health-related quality of life (HRQOL) measure derived by mapping items from the Resident Assessment Instrument – Minimum Data Set (RAI-MDS) assessment onto the Health Utilities Index Mark 2 classification system. While the validity of the MDS-HSI has been examined in cross-sectional settings, the longitudinal validity has not been explored. The objective of this study was to investigate the longitudinal construct validity of the MDS-HSI in a home care population.

**Methods:**

This study utilized a retrospective cohort of home care patients in the Hamilton-Niagara-Haldimand-Brant health region of Ontario, Canada with at least two RAI-MDS Home Care assessments between January 2010 and December 2014. Convergent validity was assessed by calculating Spearman rank correlations between the change in MDS-HSI and changes in six validated indices of health domains that can be calculated from the RAI-MDS assessment. Known-groups validity was investigated by fitting multivariable linear regression models to estimate the mean change in MDS-HSI associated with clinically important changes in the six health domain indices and 15 disease symptoms from the RAI-MDS Home Care assessment, controlling for age and sex.

**Results:**

The cohort contained 25,182 patients with two RAI-MDS Home Care assessments. Spearman correlations between the MDS-HSI change and changes in the health domain indices were all statistically significant and in the hypothesized small to moderate range [0.1 < ρ < 0.5]. Clinically important changes in all of the health domain indices and 13 of the 15 disease symptoms were significantly associated with clinically important changes in the MDS-HSI.

**Conclusions:**

The findings of this study support the longitudinal construct validity of the MDS-HSI in home care populations. In addition to evaluating changes in HRQOL among home care patients in clinical research, economic evaluation, and health technology assessment, the MDS-HSI may be used in system-level applications using routinely collected population-level data.

## Background

Generic, preference-based health-related quality of life (HRQOL) indices provide a multidimensional measure of various health domains and a single summary score of overall HRQOL and are essential for economic evaluation and health technology assessment [1]. Routine, repeated collection of preference-based HRQOL measures can enhance the capacity of health systems to assess how effectively they operate [2].

The Resident Assessment Instrument – Minimum Data Set (RAI-MDS) is a comprehensive, standardized clinical assessment with variants used in nursing homes, complex continuing care, and home and community care populations in North America (i.e., Canada and many U.S. states), Europe, and Asia/Pacific Rim [3, 4]. The Minimum Data Set Health Status Index (MDS-HSI) is a generic, preference-based HRQOL measure derived by mapping items from the RAI-MDS assessment onto the Health Utilities Index Mark 2 classification system [5]. The MDS-HSI has been previously used in both cross-sectional and longitudinal studies [6, 7].

RAI-MDS assessment variants are mandated for use in multiple care settings in many jurisdictions. As the MDS-HSI is defined using items from the RAI-MDS assessment, the MDS-HSI can be calculated on population-based, routinely collected data in jurisdictions where collection of the RAI-MDS is standard practice. Thus the MDS-HSI could be used as a standard measure to compare regularly the HRQOL of patients across settings and jurisdictions, as well as within patient trajectories in the health system. While the validity of the MDS-HSI has been examined in cross-sectional contexts, the longitudinal construct validity of the measure has not been investigated [8, 9]. Longitudinal construct validity, or responsiveness, refers to the ability of a measure to detect meaningful changes in a patient’s condition [10].

The objective of this study was to examine the longitudinal construct validity of the MDS-HSI in a large home care population using convergent and known-groups approaches. We hypothesized that change in the MDS-HSI would exhibit small to moderate negative correlations with changes in six health domain indices. The correlations were hypothesized to be negative as the indices measure increasing impairment, and in the small to moderate range as they measure a single domain of health while the MDS-HSI is a multidimensional measure. We further hypothesized that clinically important improvements or declines in health domain indices and symptoms would be associated with corresponding clinically important improvements and declines in the MDS-HSI.

## Methods

### Design and setting

This study utilized a longitudinal, population-based, retrospective cohort of adult, home care patients in the Hamilton-Niagara-Haldimand-Brant region of Ontario, Canada. This health region is one of the largest health regions in Ontario with a population of over 1.5 million persons spread across urban, suburban, and rural locales. This health region contains over 10 municipalities with wide variations in population density, socioeconomic status, and access to tertiary care centers. Community-dwelling seniors receiving publicly-funded home care in Ontario are periodically assessed with the home care variant of the RAI-MDS, the RAI-MDS Home Care, as part of their routine care.

This study involved the retrospective analysis of routinely-collected health administrative data from secondary sources; there was no primary data collection. RAI-MDS assessments that are performed as part of routine care for home care patients in Ontario are stored in the Client and Health Information System (CHRIS), which is the health administrative database used by Ontario’s publicly-funded home care agencies [11]. CHRIS additionally contains administrative data on home care episodes and billed services that can be individually linked to the clinical assessment records. CHRIS is regularly checked for validity and has been used extensively in research [12, 13]. We utilized CHRIS records stored at the Health Data Library at McMaster University to create the retrospective cohort for this study. Ethics approval was obtained from the Hamilton Integrated Research Ethics Board.

### Participants

An anonymized regional version of CHRIS was used to create a retrospective cohort of home care patients 19 years of age and older in the Hamilton-Niagara-Haldimand-Brant region with at least two RAI-MDS Home Care assessments. Administrative home care records were individually linked to clinical assessment records to identify adult patients who had a baseline assessment between January 1st, 2010 and December 31st, 2014, and a follow-up assessment between 3 and 18 months after the baseline assessment but before discharge from care. All patients 19 years of age and older with a valid baseline and follow-up assessment were included in the study.

### Measures

All measures in the study were calculated from the RAI-MDS Home Care assessment. The RAI-MDS Home Care is a comprehensive clinical assessment that contains over 200 items in the domains of function, health, social support, and service use [3]. The internationally-developed assessment was created to identify multidimensional needs, aid in the development of appropriate care plans, and establish outcomes for tracking purposes. The RAI-MDS Home Care is used for clinical home care assessment in most Canadian provinces and territories, many U.S. states, and numerous countries in Europe and Asia/Pacific Rim. RAI-MDS Home Care assessments are typically performed in-person by registered nurses and take approximately 45 minutes to complete. RAI-MDS Home Care assessment data have previously been used in clinical and epidemiological research [14]. Average inter-assessor reliability weighed kappa scores for RAI-MDS Home Care items have been estimated at 0.74 for items that are shared with the RAI MDS 2.0 and 0.70 for stand-alone home care items [15]. Pearson correlation coefficients between functional and cognitive scales from the RAI-MDS Home Care assessment and gold standard scales have been calculated to be between 0.74 and 0.81 [16]. A comparison of the RAI-MDS Home Care assessment with the International Classification of Functioning, Disability, and Health has shown substantial overlap in content [17].

The primary measure of interest in this study was change in MDS-HSI between baseline and follow-up assessment. The MDS-HSI is a preference-based HRQOL measure based on the Health Utilities Index Mark 2 classification system and defined using items from the RAI-MDS Home Care assessment. The MDS-HSI is calculated using 31 elements of the RAI-MDS Home Care assessment to map a subject onto six health domains of the Health Utilities Index Mark 2 classification system (sensation, mobility, emotion, cognition, self-care, and pain). Published community Health Utilities Index Mark 2 preference weights are applied to produce scores [5]. Previous studies have supported the validity of the MDS-HSI in cross-sectional settings, finding an intraclass correlation coefficient (ICC) between the MDS-HSI and Health Utilities Index Mark 2 of 0.46 in a home care population, increasing to 0.63 with the 10% most discrepant scores removed [5, 8]. The MDS-HSI ranges from − 0.03 to 1, with 0 representing dead and 1 perfect health. As is true with the HUI2, MDS-HSI scores less than zero represent states worse than dead. A difference of 0.03 or more indicates a clearly clinically important difference [18, 19].

The measures used in this study to assess the convergent and known-groups validity of the MDS-HSI included six validated, ordinal indices of the health domains of function, cognition, health stability, pain, mood, and communication that are calculated from RAI-MDS Home Care assessment. The ADL Hierarchy Scale measures impairment in self-performance of the activities of daily living (ADL) tasks of dressing, eating, bathing and personal hygiene [20]. The ADL Hierarchy Scale ranges from 0 (Independent) to 6 (Total Dependence). The IADL Difficulty Scale measures difficulty in performing the instrumental activities of daily living (IADL) tasks of meal preparation, housework, and telephone use [21]. The IADL Difficulty Scale ranges from 0 (no difficulty in any of the three tasks) to 6 (great difficulty in all of the tasks). The Cognitive Performance Scale (CPS) measures impairment in memory and cognitive skills [22]. CPS ranges from 0 (intact cognition) to 6 (very severe impairment). The Changes in Health, End-Stage Disease, Signs, and Symptoms Scale (CHESS) measures health instability and risk of death [23]. The CHESS scale ranges from 0 (no health instability) to 5 (highly unstable health). The Pain Scale measures the frequency and intensity of pain and ranges from 1 (no pain) to 4 (excruciating pain) [24]. The Depression Rating Scale (DRS) measures the presence and severity of mood symptoms and ranges from 0 to 14 [25]. The Communication Scale measures impairment in expression or comprehension and ranges from 0 to 8 [26]. Neither the DRS nor the Communication Scale has descriptions for specific values of the index. Also used to assess known-groups validity were 15 disease symptoms recorded on the RAI-MDS Home Care assessment: angina, constipation, dizziness, edema, dyspnea, delusions, hallucinations, dysrhythmia, poor self-reported health, flare-up of chronic condition, disruptive pain, weight loss, one or fewer meals per day, insufficient fluid intake, and declining food/liquid consumption. Age and sex were additional measures used as covariates in the linear regression analysis.

### Statistical analyses

Longitudinal construct validity was examined using convergent and known-groups approaches. Convergent validity is an aspect of construct validity that can be generated by demonstrating that the measure of interest correlates with other measures as expected [27]. In a longitudinal setting, convergent validity can be investigated by examining the correlation between the change in the measure of interest and changes in other measures with which the measure of interest is expected to be associated. In this study, we assessed convergent validity by examining the correlations between the change in the MDS-HSI and the changes in six validated indices of individual health domains. Known-groups validity is another aspect of construct validity and can be generated by demonstrating that differences exist in the measure of interest between groups that are expected to score differently [27]. In a longitudinal context, known-groups validity can be investigated by examining average change in the measure of interest in groups that are known to have experienced a change in the underlying construct. In this study, we used changes in the six health domain indices and symptoms to define patients that were known to have experienced a clinically important change in their health status and examined whether the MDS-HSI indicated that there had been a clinically important change in HRQOL.

Convergent validity was assessed using correlational methods [28–30]. The association between the MDS-HSI change scores and changes in the health domain indices were examined with Spearman rank correlations (ρ). The degree of the association was categorized according to Cohen: negligible (ρ < 0.1), small (0.1 ≤ ρ < 0.3), moderate (0.3 ≤ ρ < 0.5) and large (ρ ≥ 0.5) [31]. As the health domain indices represent single domains of health and the MDS-HSI is a multidimensional measure of HRQOL, small to moderate correlations were hypothesized. A negligible correlation would suggest that the MDS-HSI is not sensitive to change in the domain, while a large correlation would suggest that change in the domain may be inappropriately dominating change in the MDS-HSI. Given that the health domain indices measure increasing impairment on their individual domains, the correlations were hypothesized to be negative.

Known-groups validity was assessed using linear regression to estimate the mean change in MDS-HSI associated with a clinically important change in each of the health domains or symptoms while controlling for age and sex [30]. A clinically important change in any of the health domain indices or symptoms represents a change in the patient condition which would generally be expected to be associated with a clinically important change in HRQOL. Multivariable linear regression models were fit for each index and symptom with change in MDS-HSI as the dependent variable and change in the index or symptom as an independent variable. One-unit changes were taken to be clinically important for all indices except the depression rating scale, where, by convention, a two-unit change is considered clinically important. Minimal clinically important difference ratios were calculated by dividing the regression estimates of mean change in MSI-HSI by 0.03. All models included age and sex as covariates with age treated as a linear continuous variable.

Linear regression was selected as the analysis method as the distribution of the change in MDS-HSI was expected to be reasonably close to the normal distribution (Appendix). Models were checked for multicollinearity and violations of model assumptions by examining variance inflation factors and residual and diagnostic plots. Cases with missing data were removed from the analysis. All analyses were performed using SAS 9.4 (SAS Institute, Cary NC).

### Sensitivity analysis

Several sensitivity analyses were undertaken to investigate the robustness of results. These included restricting the follow-up assessment window to be between 6 and 12 months after the initial assessment rather than between 3 and 18 months, stratifying analyses by sex rather than adjusting for sex, using Pearson correlations rather than Spearman correlations, altering the formulation of the age covariate, and excluding age and sex from the models.

## Results

### Descriptive characteristics

**Fig. 1:**
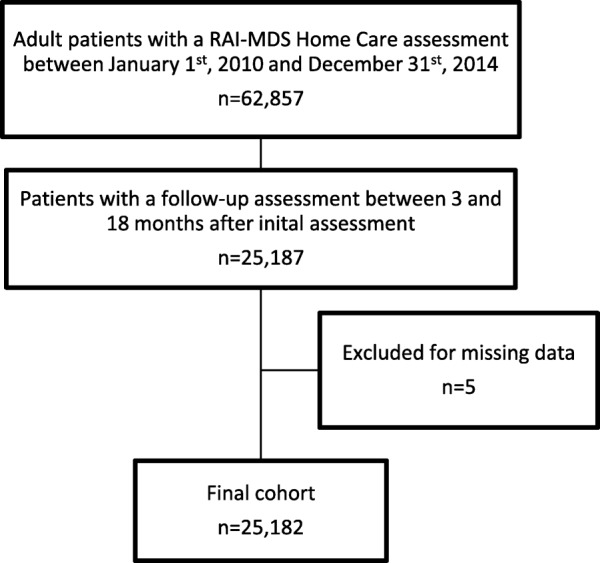
Flow diagram of cohort creation

There were 62,857 adult home care patients in the Hamilton-Niagara-Haldimand-Brant region with a baseline RAI-MDS Home Care assessment between January 1st, 2010 and December 31st, 2014 (Fig. 1). Of those patients, 25,187 received a follow-up RAI-MDS Home Care assessment between 90 and 540 days after the baseline assessment without being discharged from care between the assessments. Five cases (0.02%) had missing data and were excluded from the analysis, reducing the final cohort to 25,182.

The characteristics of the study cohort can be found in Table 1. The participants tended to be elderly (78 ± 14 years) and nearly two-thirds were female (65.6%). Just over one-third (35.1%) lived alone. Significant impairments in the activities of daily living (45.4%) and cognitive performance (60.9%) were common, as well as mood symptoms (39.0%) and a recent history of falls (41.5%). A large majority of patients (83.6%) had multiple chronic conditions. The mean baseline MDS-HSI was 0.50, falling to 0.48 at follow-up. Between the baseline and follow-up assessments, 9872 (39.2%) patients experienced a decline in MDS-HSI, 8767 (34.8%) had no change, and 6543 (26.0%) improved.

**Table 1:** Characteristics of participants in the study

Patient characteristics	No. (%)
	*n* = 25,182
Demographic Characteristics	
Age (Mean ± SD)	77.91 ± (13.54)
Sex (F)	16,512 (65.6%)
Lives Alone	8846 (35.1%)
Health Characteristics	
ADL Impairment^a^	11,436 (45.4%)
Cognitive Impairment^b^	15,431 (60.9%)
Dyspnea	6440 (25.6%)
Poor Self-Reported Health	4806 (19.1%)
Fall in last 90 Days	10,447 (41.5%)
Mood Symptoms^c^	9831 (39.0%)
Wandering	521 (2.1%)
Aggressive Behaviour^d^	1920 (7.6%)
Weight Loss^e^	2098(8.3%)
Number of Medications (Mean ± SD)	7.41 ± (2.30)
Informal Caregiver Status	
Caregiver express distress^f^	4568 (18.1%)
Information Care Hours per Day (Mean ± SD)	2.67 ± (2.99)
Diagnoses	
Cardiovascular	12,629 (50.2%)
Dementia	5628 (22.3%)
Neurological	2526 (10.0%)
Musculoskeletal	16,662 (66.2%)
Psychiatric	4591 (18.2%)
Cancer	2818 (11.2%)
Diabetes	6542 (26.0%)
COPD	4379 (17.4%)
Count of Chronic Conditions^g^	
0	888 (3.5%)
1	3249 (12.9%)
2	5629 (22.4%)
3	6395 (25.4%)
4	4675 (18.6%)
5	2639 (10.5%)
6	1145 (4.6%)
7+	562 (2.2%)

^a^ ADL Hierarchy Scale > 0

^b^ Cognitive Performance Scale > 0

^c^ Depression Rating Scale > 0

^d^ Verbal abuse, physical abuse, socially inappropriate behaviour, or resistance to care in last 3 days

^e^ Unintended weight loss of 5% or more in the last 30 days or 10% or more in the last 180 days

^f^ Caregiver expresses feelings of distress, anger, or depression

^g^ Includes: Stroke, congestive heart failure, coronary artery disease, hypertension, dementia, Parkinson’s disease, multiple sclerosis, arthritis, osteoporosis, psychiatric diagnosis, cancer, diabetes, emphysema, and renal failure

### Convergent validity

Spearman rank correlation coefficients (ρ) between the MDS-HSI change scores and health domain change scores are presented in Table 2. MDS-HSI change scores were significantly correlated with changes in each of the health domain indices, ranging from − 0.48 [− 0.49,-0.47] for the pain scale to − 0.21 [− 0.23,-0.20] for the communication scale. All correlations were negative and in the small to moderate range.

**Table 2:** Spearman correlation (ρ) between change in health domain indices and change in MDS-HSI

Health Domain Index	ρ (95% CI)	*p*-value
Change in ADL Hierarchy (Function)	− 0.375 (− 0.385, − 0.364)	<.0001
Change in IADL Difficulty (Function)	− 0.287 (− 0.298, − 0.276)	<.0001
Change in CPS (Cognition)	− 0.281 (− 0.292, − 0.269)	<.0001
Change in CHESS (Health Stability)	− 0.331 (− 0.342, − 0.320)	<.0001
Change in DRS (Mood)	− 0.380 (− 0.391, − 0.370)	<.0001
Change in Communication Scale	− 0.213 (− 0.225, − 0.201)	<.0001
Change in Pain Scale	−0.482 (− 0.492, − 0.473)	<.0001

### Known-groups validity

Results from the regression analysis can be found in Table 3. The mean change in MDS-HSI associated with clinically important changes in the health domain indices exceeded the minimal clinically important difference of 0.03 in each case. Minimal clinically important difference ratios varied from 1.27 for the communication scale to 3.04 for the pain scale.

**Table 3:** Regression estimates of mean change in MDS-HSI by clinically important changes in indices and symptoms

Measure	Mean change in MDS-HSI (β) (95% CI)^a^	*p*-value	MCID Ratio^b^
Health Domain Index			
ADL Hierarchy (Function)	−0.055 (− 0.057, − 0.053)	<.0001	1.84
IADL Difficulty (Function)	− 0.045 (− 0.047, − 0.043)	<.0001	1.50
CPS (Cognition)	− 0.065 (− 0.068, − 0.062)	<.0001	2.16
CHESS (Health Stability)	− 0.050 (− 0.052, − 0.049)	<.0001	1.68
DRS (two steps) (Mood)	− 0.066 (− 0.069, − 0.064)	<.0001	2.21
Pain Scale	− 0.091 (− 0.093, − 0.089)	<.0001	3.04
Communication Scale	− 0.038 (− 0.040, − 0.036)	<.0001	1.27
Symptom			
Angina	− 0.030 (− 0.040, − 0.019)	<.0001	1.00
Constipation	− 0.057 (− 0.073, − 0.042)	<.0001	1.91
Dizziness	− 0.039 (− 0.044, − 0.033)	<.0001	1.28
Edema	− 0.028 (− 0.033, − 0.022)	<.0001	0.92
Dyspnea	− 0.032 (− 0.038, − 0.026)	<.0001	1.07
Delusions	− 0.081 (− 0.097, − 0.066)	<.0001	2.71
Hallucinations	− 0.084 (− 0.097, − 0.071)	<.0001	2.79
Dysrhythmia	− 0.016 (− 0.029, − 0.002)	0.022	0.52
Poor self-reported health	− 0.068 (− 0.075, − 0.061)	<.0001	2.28
Flare-up of chronic condition	− 0.062 (− 0.070, − 0.055)	<.0001	2.08
Disruptive Pain	− 0.140 (− 0.145, − 0.135)	<.0001	4.68
Weight Loss	− 0.063 (− 0.070, − 0.057)	<.0001	2.11
One or fewer meals per day	− 0.076 (− 0.087, − 0.064)	<.0001	2.52
Insufficient fluid intake	− 0.086 (− 0.102, − 0.069)	<.0001	2.86
Declining food/liquid consumption	− 0.110 (− 0.121, − 0.099)	<.0001	3.67

^a^ Estimates correspond to an increase in the health domain index (increasing impairment) or new onset of a symptom

^b^ MCID = β / 0.03

Of the 15 symptoms examined, changes in 13 of them were associated with a clinically important change in the MDS-HSI. The greatest change was observed with the resolution or onset of pain intense enough to disrupt activities (minimal clinically important difference ratio 4.68), and the smallest change was associated with resolution or onset of dysrhythmia (minimal clinically important difference ratio 0.52). No variance inflation factors over 5 were observed and examination of residual and diagnostic plots did not indicate any substantial departures from model assumptions.

### Sensitivity analyses

Restricting the population to only those with a valid follow-up assessment between 6 and 12 months after the initial assessment had no meaningful impact on the results (Tables 4 and 5). Stratifying results by sex produced very similar results for both males and females. Alternative formulations of age as a cubic smoothing spline or the inclusion of higher-order age polynomials had no significant impact on the model. The overall exclusion of age and sex from the models likewise had no significant impact.

**Table 4:** Sensitivity analysis of convergent validity results

Health domain index	Spearman rank correlation coefficients (ρ)			
	Study population	6 to 12 month follow-up window	Male only	Female only
	*n* = 25,182	*n* = 14,676	*n* = 8670	*n* = 16,512
Change in ADL Hierarchy (Function)	− 0.375	− 0.386	− 0.385	− 0.368
Change in IADL Difficulty (Function)	− 0.287	− 0.291	− 0.262	− 0.300
Change in CPS (Cognition)	− 0.281	− 0.302	− 0.304	− 0.267
Change in CHESS (Health Stability)	− 0.331	− 0.322	− 0.332	−0.330
Change in DRS (Mood)	−0.380	−0.372	− 0.371	−0.385
Change in Communication Scale	−0.213	−0.229	− 0.217	−0.210
Change in Pain Scale	−0.482	−0.480	− 0.476	−0.486

All correlations were statistically significant at α = 0.001

**Table 5:** Sensitivity analysis of known-groups validity results

Measure	Regression estimates (β) of Mean Change in MDS-HSI			
	Study population	6 to 12 month follow-up window	Male only	Female only
	*n* = 25,182	*n* = 14,676	*n* = 8670	*n* = 16,512
Health domain index				
ADL Hierarchy (Function)	−0.055	−0.056	− 0.057	−0.054
IADL Difficulty (Function)	−0.045	−0.045	− 0.045	−0.045
CPS (Cognition)	−0.065	−0.068	− 0.068	−0.063
CHESS (Health Stability)	−0.050	−0.049	− 0.050	−0.051
DRS (two steps) (Mood)	−0.066	−0.065	− 0.072	−0.064
Pain Scale	−0.091	−0.091	− 0.086	−0.094
Communication Scale	−0.038	−0.041	− 0.038	−0.038
Symptom				
Angina	−0.030	− 0.024	−0.039	− 0.025
Constipation	−0.057	− 0.061	−0.055	− 0.058
Dizziness	−0.039	− 0.039	−0.037	− 0.039
Edema	−0.028	− 0.026	−0.022	− 0.030
Dyspnea	−0.032	− 0.028	−0.036	− 0.030
Delusions	−0.081	− 0.081	−0.087	− 0.078
Hallucinations	−0.084	− 0.083	−0.089	− 0.081
Dysrhythmia	−0.016	− 0.013^*^	− 0.008^*^	− 0.020
Poor self-reported health	−0.068	− 0.061	−0.076	− 0.064
Flare-up of chronic condition	−0.062	− 0.060	−0.068	− 0.060
Disrupting Pain	−0.140	− 0.139	−0.137	− 0.142
Weight Loss	−0.063	− 0.058	−0.068	− 0.060
One or fewer meals per day	−0.076	− 0.071	−0.070	− 0.078
Insufficient fluid intake	−0.086	− 0.083	−0.096	− 0.081
Declining food/liquid consumption	−0.110	− 0.105	−0.109	− 0.110

^*^ *p* > 0.05

All other parameter estimates were statistically significant at α = 0.05

## Discussion

The longitudinal construct validity of an instrument is an important property to consider for any application that involves measuring change across multiple time points. This study investigated the longitudinal construct validity of the MDS-HSI with respect to changes in individual health domains and disease symptoms in adult, home care patients in Ontario, Canada. The authors are not aware of any previous study into the responsiveness of the MDS-HSI even though the measure has been used in longitudinal studies [6].

This investigation into the longitudinal convergent and known-groups validity of the MDS-HSI supports the responsiveness of the measure. Correlation between MDS-HSI change scores and change scores of the health domain indices were within expected ranges and indicated that the MDS-HSI is sensitive to changes in all of the domains examined. Regression analysis showed that clinically important changes in all of the indices and in 13 of the 15 symptoms were associated with clinically important changes in the MDS-HSI, indicating that the measure is able to detect differences in HRQOL among groups that are known to have changed.

Like other validated HRQOL measures, the MDS-HSI can be used in clinical research, economic evaluation, and health technology assessment. However, the ability of the MDS-HSI to be calculated from routinely-collected population-level data suggests additional possibilities and applications. Typically, economic evaluation requires a prospective study incorporating an HRQOL survey. However, because the MDS-HSI can be calculated from data that is already stored in administrative health databases in many jurisdictions, there is the potential that population-level economic evaluation could be performed retrospectively. Additionally, regular measurement of HRQOL using a tool such as the MDS-HSI could produce benefits on both the patient and system level. For example, regular collection of a preference-based HRQOL measure for patients with chronic diseases would allow clinicians and patients to develop care plans focused on improving the issues that are the most important to the patient. At the system level, regular collection of HRQOL data would enable a more general measurement of health system performance, providing a standard quality measurement across sectors and identifying key areas for improvement. The MDS-HSI would be an ideal instrument for regular measurement of HRQOL in care settings that routinely collect the RAI-MDS as no additional assessments would be required. As RAI-MDS assessment variants are used in numerous countries in North America, Europe, and Asia/Pacific Rim, the MDS-HSI could be used to measure and compare the HRQOL of millions of patients around the world with minimal change in practice.

### Limitations

The limitations of this study include lack of access to an external gold standard criterion and uncertainty of the generalizability of the results outside of home care settings. Additional research should be conducted to continue to investigate the validity of the MDS-HSI, in particular, the comparability of results between different care settings.

## Conclusion

The results of this study support the longitudinal construct validity of the MDS-HSI in home care populations. Analysis using convergent and known-groups approaches suggests that the MDS-HSI is responsive to changes in a broad array of health domains and disease symptoms. These findings support the usage of the measure to evaluate changes in HRQOL among home care patients in clinical research, economic evaluation, and health technology assessment. In addition, the calculability of the MDS-HSI from routinely collected data suggests other applications such as system-level performance measurement and use in retrospective studies.

## Figures and Tables

**Fig. 2:**
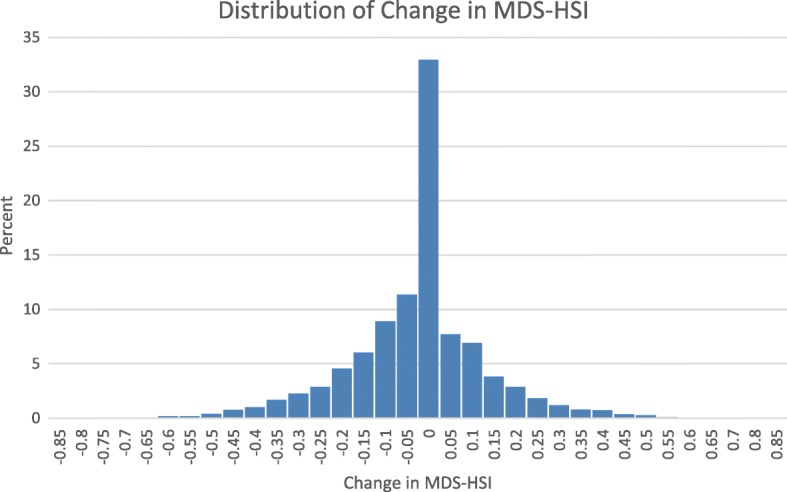
Distribution of change in MDS-HSI

## Abbreviations

ADL: Activities of daily living
CHESS: Changes in health, end-stage disease, signs and symptoms scale
CHRIS: Client and health information system
CPS: Cognitive performance scale
DRS: Depression rating scale
HRQOL: Health-related quality of life
IADL: Instrumental activities of daily living
ICC: Intraclass correlation coefficient
MDS-HSI: Minimum data set health status index
RAI-MDS: Resident assessment instrument – minimum data set
